# Naltrexone as a Novel Therapeutic for Diabetic Corneal Complications

**DOI:** 10.33696/immunology.1.018

**Published:** 2020

**Authors:** Patricia J. McLaughlin, Joseph W. Sassani, Ian S. Zagon

**Affiliations:** 1 Department of Neural and Behavioral Sciences, Penn State University College of Medicine, Hershey, Pennsylvania, 17033, USA; 2 Department of Ophthalmology, Penn State University College of Medicine, Hershey, Pennsylvania, 17033, USA

**Keywords:** Diabetes, Corneal surface abnormalities, Dry eye, Naltrexone

## Abstract

Diabetes is a widespread autoimmune disorder that affects nearly 10% of the adult population in the United States. In addition to the primary disease, there are numerous complications associated with inflammation including abnormalities of the heart, visual system, and peripheral nervous system. More than half of the individuals with diabetes will have one or more ocular related complications such as dry eye disease (DED), keratopathy, or retinopathy. Research over the last 3 decades has focused on the role of the opioid growth factor – opioid growth factor receptor (OGF-OGFr) axis as a regulatory system that maintains homeostasis in corneal epithelialization and tear secretion. In diabetes, OGF appears to be dysregulated resulting in decreased cell replication and increased corneal surface sensitivity. Utilization of naltrexone as a topical therapeutic to block the OGF-OGFr axis results in reversal of dry eye and restoration of corneal sensitivity and rates of corneal re-epithelialization. Naltrexone treatment at dosages that are substantially lower than systemically approved doses appear to be safe and effective therapy for corneal surface abnormalities associated with diabetes.

## Introduction

### Diabetes and corneal complications

Diabetes is approaching epidemic proportions worldwide. In the United States, there are more than 30 million individuals having a confirmed diagnosis of diabetes [1,2]. Worldwide, the number of people with diabetes exceeds 400 million [3], and is expected to reach 550 million by 2030. Diabetes is associated with complications that affect nearly all systems, including vision. While retinopathy and diabetic cataracts receive the most attention, more than 54% of diabetic individuals will experience at least one complication related to ocular surface disease including keratopathy, dry eye, and aberrant sensitivity [4]. *Keratoconjuntivitis sicca*, dry eye syndrome, may be either symptomatic or asymptomatic in individuals with diabetes depending upon the status of corneal sensory function [5]. Additionally, reduction in corneal epithelial adhesion complexes required for complete epithelialization of epithelial defects and reduced density of the stromal nerve network may lead to recurrent epithelial injury and incomplete corneal surface healing resulting in recurrent erosions. Any or all of these abnormal characteristics can be associated with diabetes [6–11]. Thus, there is an unmet medical need to identify effective treatments targeting corneal complications associated with diabetes.

### Current therapies

The first line current therapy for dry eye only includes many over-the-counter (OTC) drops such as Systane®, Thera Tears®, Blink®, and Soothe®. The majority of OTC drops are lubricants that must be administered frequently throughout the day and are most effective for episodic dry eye caused by dry air, smoke, or other adverse environmental conditions. The two most widely used FDA approved prescription therapies are cyclosporine (Restasis®) and Lifitegrast (Xiidra®) [12,13]. Restasis® requires a lengthy period (weeks) of application before symptoms are reduced. Xiidra® has recently received FDA approval to reduce inflammation. Both compounds are expensive and do not address the underlying cause of diabetic dry eye. Feedback from clinical reports supports the need for effective, less expensive therapeutics [14].

### Role of endogenous opioids and opioid receptors in ocular disease

Our laboratory made the discovery more than four decades ago that an endogenous opioid, specifically opioid growth factor (OGF), chemically termed [Met^5^]-enkephalin, interacted with a nuclear receptor to function in the homeostasis of cellular replication [15]. Upon isolation and cloning of the receptor, it was determined to be unlike other opioid receptors, and was named opioid growth factor receptor (OGFr) [16]. OGF is an inhibitory growth factor that is autocrine and paracrine produced and targets normal and abnormal replicating tissues. OGF inhibits the cell cycle by upregulating cyclin-dependent inhibitory kinases prior to the S phase of the cell cycle in both neoplasia [17] and normal tissues [18]. In adult subjects, OGF and OGFr expression are robust in tissues that are constantly renewing such as the corneal epithelium, skin, and gastrointestinal track. OGF and OGFr expression have been recorded in corneal tissues of a variety of normal vertebrates [19], and the nucleotide sequence is found in over 1400 animal species, 250 fungi, and 800 bacteria (see https://www.ncbi.nlm.nih.gov/nuccore/?term=OGFr). The OGF-OGFr axis can be blocked by opioid antagonists such as naloxone and naltrexone. Several studies from our laboratory demonstrated that the duration of receptor blockade is more important than dosage [15]. Both naloxone and naltrexone bind to the nuclear associated OGFr, but naltrexone has greater affinity and has been used in most of our studies. If the naltrexone blockade of OGFr is complete over a 24-hr interval between daily dosing, the result is accelerated DNA synthesis and cellular replication. If the blockade is intermittent, as can be produced by low doses of naltrexone, the result is decreased DNA synthesis. Hence, the OGF-OGFr relationship represents a novel regulatory pathway that can be easily modulated for the treatment of corneal surface complications in diabetes.

### Diabetes and endogenous opioids and opioid receptors

The findings from several decades of research on the cornea are summarized by Sassani et al. [20] and demonstrate that blockade of the OGF-OGFr regulatory pathway with naltrexone provides an effective and safe treatment for some ocular surface complications of diabetes [21–26]. Based on our knowledge that naltrexone blockade of the OGF-OGFr axis accelerates diabetic corneal epithelial healing in rats and mice, and the knowledge that human diabetics experience dry eye [4], our laboratory began to investigate restoration of tear production in normal rats [27 and rodent models of diabetes [26,28]. Our hypothesis-based research was supported by the knowledge that serum levels of [Met^5^]-enkephalin are elevated in individuals with diabetes [29]. Thus, we hypothesized that dysregulation of the OGF-OGFr axis in diabetes leading to increased expression of OGF and/or its receptor may inhibit cellular proliferation and/or innervation in the cornea that is required for normal tear production.

### Novel therapy – opioid growth factor receptor antagonists for treatment of corneal surface complications

Because of the prevalence of keratopathy and dry eye in diabetic individuals [4], our laboratory focused on these abnormalities in rat models of type 1 diabetes (T1D) and began studying blockade of the OGF-OGFr regulatory axis with naltrexone. Specifically, utilizing a standard streptozotocin (STZ)-induced model of T1D, it was documented that T1D rats have delayed corneal surface healing rates relative to normal animals [22–24]. Corneal epithelial cell DNA synthesis rates are decreased in T1D by more than 90% compared to control levels. Dose-range studies (10-4 to 10-6 M) of naltrexone administered topically to the corneal surface 4 times/day for 7 days resulted in 25% to 83% smaller epithelial defects in T1D rats. This reduction (~33%) in corneal wound closure time in T1D rats receiving topical naltrexone corresponded to 20% to 42% faster healing rates compared to type 1 diabetes (T1D) animals treated only with vehicle. In fact, T1D animals receiving 10-5 M NTX were comparable to normal animals in all parameters of corneal wound closure. Studies were extended to type 1 diabetic rabbits induced with alloxan [25] and to the genetic db/db mouse model of type 2 diabetes (T2D) [26]. In all animal models of diabetes, topical naltrexone was effective at restoring the rate of corneal epithelial wounds to normal levels. In these studies, serum or tissue levels of OGF or OGFr were not determined, and because of IACUC limitations on maintaining animals with uncontrolled T1D, peptide and receptor expression levels in early and late stages of diabetes have not been systematically reported.

### Preclinical studies on use of topical opioid antagonist as a novel therapeutic for dry eye

Encouraged by these results and given the fact that more than 20 million individuals in the United States and 50 million worldwide have dry eye [4], we extended our studies to examine the efficacy of topical naltrexone to reverse diabetic dry eye disease. Using rat and mouse models of T1D and T2D, respectively, we demonstrated that topical application of naltrexone dissolved in moxifloxacin (Vigamox®) eye drops restored corneal surface sensitivity and tear fluid volume [26,28]. A single eye drop of naltrexone resulted in normal tear fluid measurements (i.e., reversed dry eye) within hours. Corneal surface sensitivity was measured using an aesthesiometer. Dry eye was evaluated using the Schirmer l test whereby Schirmer paper strips are held inside the lower lid cul-de-sac and the wetting length measured to the nearest half millimeter. Topical naltrexone did not result in any overt toxicity (changes in intraocular pressures, corneal thickness, endothelial cell number, epithelial apoptosis/necrosis) in the treated eye, and had no adverse effect on the untreated eye in either the rat or mouse model of diabetes.

### Novel topical naltrexone as a therapeutic for dry eye

Based on our data cited above, translational studies were conducted with a formulation commercially prepared under good laboratory practice (GLP) standards containing 20 μg/ml naltrexone in a proprietary vehicle carrier [30]. T1D rats received one drop twice daily of the active formulation for 10 days, whereas controls received only the vehicle. A single drop of the proprietary formulation increased tear volume within 4 hr in comparison to the baseline tear volume for each rat. The vehicle control had no effect on tear volume; whereas in the active group, there was a sustained reversal of dry eye measurements to normal levels throughout the 10-day study. Furthermore, corneal sensitivity as measured using the Cochet-Bonnet aesthesiometer was restored following naltrexone treatment. Diabetes-induced corneal insensitivity in rats was evident because twice the force was needed to elicit a response as required by normal rats. Within 48 hr of application of the proprietary formulation, corneal sensitivity in the treated eye of the T1D rats was restored to levels recorded in normal rats. Tear fluid “wetting’ measurements differed significantly from those of T1D baseline and T1D rats receiving buffer (p<0.0001). The dosage of naltrexone (20 μg/ml) did not invoke changes to intraocular pressure, and the effects of normal tear production appeared to last up to 96 hr following termination of naltrexone treatment.

Safety and toxicity were tested in male (126-150 g) Sprague-Dawley rats (Charles River Laboratories) and male 2 kg New Zealand white rabbits purchased from Robinson Services Inc (RSI) [30]. In the safety study, the right eye of each animal was treated twice daily with two drops at each dosing of either naltrexone eye drops or vehicle eye drops. Thus, each eye received twice the dose used to measure efficacy which was a single eye drop twice daily. The length of treatment in the safety study was 30 days in comparison to the 10-day treatment evaluated for efficacy of naltrexone dosages. Both right and left eyes were processed for histopathology and slides were assessed in a masked manner by a third-party veterinary ophthalmic pathologist. No evidence of inflammatory, neoplastic or degenerative change was noted in any rat eye. Qualitative data based on observations by two graduate assistants who administered formulations twice daily, as well as the veterinarians and animal handling technicians who were observing the animals at least once daily, revealed that there were no signs of distress immediately following application (e.g., squealing, crying, tearing, or vocalizations), and no redness or crusting in the treated eye was noted by the technicians. At the termination of the 30-day study, both rat eyes appeared clear (no cataracts), with no excessive tearing.

The pathologic report for the rabbit eyes indicated that there was no evidence of neoplastic or degenerative change [30] following naltrexone treatment. Qualitative assessments were made by the veterinary technicians who were treating the rabbits twice daily for 30 days and reported no eye abnormalities such as redness, edema, irritation, or excess tearing or dryness. The rabbits displayed no signs of pain or distress upon administration of the eye drops or immediately following administration.

In summary, investigations of the novel GLP-compliant naltrexone formulation was effective at blocking the OGF-OGFr axis for sufficient duration to reverse dry eye and restore corneal surface sensitivity in diabetic rats, and sustained application for 30 days revealed no toxicity in normal rats and rabbits. Thus, the evidence from numerous preclinical studies [22–26] supported the appropriateness of performing preliminary human studies.

### Clinical studies on use of topical opioid antagonist in humans

A small clinical study approved by the Penn State Institutional Review Board and funded by the Department of Defense was completed and demonstrated tolerability of naltrexone dissolved in Vigamox® [31]. Subjects received eyedrops containing increasing dosages of naltrexone that were applied 4 times daily over a 24 hr period of time. No adverse events, clinical pathology, or changes in vision were noted. After the preclinical study on the propriety formulation was completed [30], an IND application was submitted to continue human trials.

The FDA approved an exploratory trial to examine safety and efficacy of 0.002% naltrexone ophthalmic solution relative to placebo in 60 diabetic subjects for 1 month, 30 of whom received eyedrops containing the active ingredient and 30 received vehicle. The study was conducted by a contract research organization that had familiarity in recruiting diabetic patients with dry eye disease. Inclusion criteria required that subjects had either type 1 or type 2 diabetes with a reported history of dry eye for at least 6 months. Exclusion criteria were extensive in order to avoid subjects with uncontrolled diabetes and/or potentially confounding eye disease. Endpoints included 9 factors such as fluorescein staining, lissamine green staining, tear film break-up time, tear osmolarity, and Schirmers Test without anesthesia. Additional endpoints included ocular discomfort and assessments of dryness, burning, stinging, and grittiness. Safety endpoints included visual acuity, slit-lamp biomicroscopy, intraocular pressure, and dilated fundoscopy information. Data were evaluated as population data using Modified Intent-to-Treat Population (mITT) – The mITT population included all randomized subjects with at least one post-Visit 2 trial assessment. Subjects in the mITT population were analyzed as randomized

The results supported and extended the safety record for the compound. All of the recruited patients completed the 30-day trial. Because the trial was exploratory in nature, it was not powered to show statistical differences for any efficacy measure.

Conclusions of the clinical trial have been registered with the ClinTrials.gov. Regarding efficacy, consistent improvements in naltrexone-treated subjects compared to vehicle-treated subjects were observed throughout the trial. More importantly, statistically significant improvements were observed for several symptomatic measures. The overall trends suggest a potential therapeutic benefit from the naltrexone therapy. Novel formulations of naltrexone may also confirm efficacy and use of this therapeutic [32]. The contract research organization reported that “based upon the criteria established to assess safety, by all measures it is safe for topical instillation”. There were no treatment-associated changes observed and only one ocular-related treatment-emergent adverse event which was not clinically significant. Comparisons of active versus vehicle, and active versus baseline data, demonstrated positive trends for naltrexone in relief of DED, and reported examples of statistically significant improvements in symptomatic measures.

## Conclusions

Preclinical and clinical data support the use of a novel formulation containing naltrexone as a therapeutic modality for treatment of ocular surface complications associated with diabetes including delayed corneal epithelialization following injury, hypersensitivity, and DED. Additional studies are planned to assess efficacy of higher dosages of naltrexone utilized over longer periods of time, as well as additional safety and tolerability studies in diabetic individuals.
